# Advances in Polymeric Drug Delivery Systems

**DOI:** 10.3390/pharmaceutics18091058

**Published:** 2026-08-26

**Authors:** Barbara Zawidlak-Węgrzyńska, Joanna Rydz

**Affiliations:** 1 Department of Chemistry, Faculty of Medicine in Zabrze, Academy of Silesia, 40-555 Katowice, Poland; 2 Centre of Polymer and Carbon Materials, Polish Academy of Sciences, M. Curie-Skłodowska 34, 41-819 Zabrze, Poland; jrydz@cmpw-pan.pl

Polymeric drug delivery systems continue to play a pivotal role in modern pharmaceutical research, offering innovative solutions for overcoming limitations associated with conventional drug administration. Advances in polymer science, nanotechnology, and biomaterials engineering have enabled the development of sophisticated delivery platforms capable of improving drug stability, bioavailability, targeting efficiency, and controlled release behavior. The growing interest in biodegradable and biocompatible polymers, together with emerging fabrication technologies such as additive manufacturing, has further expanded the possibilities for designing personalized and highly effective therapeutic systems [1,2,3,4,5,6]. Over the past decade, polymer-based drug delivery technologies have evolved from relatively simple sustained-release formulations to multifunctional systems capable of responding to biological and environmental stimuli. Modern polymeric carriers can be engineered to release therapeutic agents in response to changes in pH, temperature, enzyme activity, redox potential, or external physical stimuli such as light, magnetic fields, and ultrasound. Such stimuli-responsive platforms have demonstrated considerable potential for improving therapeutic precision while reducing systemic toxicity, particularly in the treatment of cancer, inflammatory disorders, and infectious diseases [7,8,9]. Natural polymers, including gelatin, chitosan, alginate, hyaluronic acid, collagen, and cellulose derivatives, continue to attract significant attention because of their excellent biocompatibility in various biological environments, biodegradability, and intrinsic biological activity. At the same time, synthetic polymers such as poly(lactic-*co*-glycolic acid) (PLGA), poly(ethylene glycol) (PEG), polycaprolactone (PCL), and poly(*N*-isopropylacrylamide) (PNIPAM) provide superior control over physicochemical properties, degradation kinetics, and drug release profiles. Hybrid systems combining natural and synthetic polymers are increasingly being developed to exploit the complementary advantages of both material classes, resulting in delivery platforms with improved mechanical properties, stability, and therapeutic performance [2,4,5]. Recent advances in nanotechnology have further accelerated the development of polymeric nanoparticles, polymeric micelles, dendrimers, nanogels, hydrogels, electrospun nanofibers, and implantable drug delivery devices. These systems enable the encapsulation of a broad spectrum of therapeutic agents, including small-molecule drugs, proteins, peptides, nucleic acids, vaccines, and gene-editing components. The rapid clinical success of lipid nanoparticle-based RNA therapeutics has also stimulated renewed interest in polymeric nanocarriers as versatile alternatives capable of achieving enhanced stability, prolonged circulation, targeted delivery, and controlled intracellular release [3,4,8]. Another important trend is the integration of advanced manufacturing technologies with polymer science. Three-dimensional printing, microfluidics, electrospinning, and biofabrication techniques have created new opportunities for producing patient-specific dosage forms and implantable devices with precisely controlled architecture and drug distribution. Combined with computational modeling, artificial intelligence, and machine learning approaches for formulation optimization, these technologies are expected to substantially accelerate the development of next-generation polymeric drug delivery systems and facilitate their translation into clinical practice [6,9]. Despite remarkable progress, several challenges remain before many experimental polymeric delivery platforms achieve widespread clinical implementation. These include large-scale manufacturing, reproducibility, long-term safety, regulatory approval, and cost-effective production processes. Addressing these issues requires close collaboration among materials scientists, chemists, pharmacists, biomedical engineers, clinicians, and regulatory experts. Continued interdisciplinary research is therefore essential for translating innovative polymer-based delivery technologies into safe, effective, and clinically accessible therapeutic solutions capable of addressing unmet medical needs across a wide range of diseases. The Special Issue “Advances in Polymeric Drug Delivery Systems” was launched to provide a platform for the dissemination of recent developments in the design, synthesis, characterization, and application of polymer-based drug delivery technologies. The published contributions cover a broad range of topics, from fundamental material design to advanced therapeutic applications.

Several review articles provide comprehensive overviews of emerging materials and delivery strategies. Milano et al. reviewed current trends in gelatin-based drug delivery systems, highlighting the versatility of gelatin as a natural polymer and its applications in various therapeutic formulations [10]. Garcia et al. examined recent patents related to nanocellulose in pharmaceutical applications, emphasizing the growing interest in sustainable and renewable biomaterials for drug delivery [11]. Liu et al. discussed hydrogel-based therapeutic approaches for pancreatic ductal adenocarcinoma, focusing on the ability of hydrogels to improve localized treatment and modulate the tumor microenvironment [12]. In another contribution, Yu et al. summarized recent advances in smart polymeric nanoparticles for cancer immunotherapy, illustrating how stimuli-responsive nanocarriers can enhance therapeutic efficacy and immune modulation [13].

The Special Issue also includes reviews addressing specialized drug delivery applications. Rathi et al. present a comprehensive analysis of rectal drug delivery systems, including clinical and patent perspectives, demonstrating the continuing relevance of this route for both local and systemic drug administration [14]. Zawidlak-Węgrzyńska et al. reviewed polymer–drug anti-thrombogenic and hemocompatible coatings designed to reduce thrombotic complications associated with blood-contacting medical devices [15].

Original research articles further illustrate the diversity and innovation of contemporary polymeric drug delivery research. Domiński et al. developed a supramolecular hydrogel system based on α-cyclodextrin and pH-responsive micelles for the co-delivery of 8-hydroxyquinoline glycoconjugates and doxorubicin, demonstrating enhanced antitumor potential through controlled and synergistic drug release [16]. Joiner et al. investigated the influence of drug physicochemical properties on polymer degradation and release kinetics in in situ forming implants, providing important insights into formulation design for long-acting delivery systems [17]. Gohn et al. examined the dissolution behavior of active ingredients incorporated into ethylene vinyl acetate (EVA) copolymer implants, highlighting the impact of particle size and morphology on release performance [18].

Collectively, the contributions published in this Special Issue demonstrate the rapid evolution of polymeric drug delivery technologies and their expanding role in addressing current pharmaceutical and biomedical challenges. The studies presented herein underscore the importance of interdisciplinary collaboration among materials scientists, chemists, pharmacists, engineers, and clinicians in translating innovative delivery concepts into practical therapeutic solutions.

We sincerely thank all authors for their valuable contributions, the reviewers for their critical assessments and constructive comments, and the editorial staff of *Pharmaceutics* for their professional support throughout the publication process. We hope that this Special Issue will contribute to further advances in polymeric drug delivery research and stimulate future developments in this important field.

## Data Availability

No new data were created or analyzed in this study. Data sharing is not applicable to this article.
